# Introduction to Pain Management for Third-Year Medical Students Team-Based Learning Module

**DOI:** 10.15766/mep_2374-8265.11095

**Published:** 2021-02-11

**Authors:** Kiernan Smith

**Affiliations:** 1 Assistant Professor, Department of Family and Community Medicine, Tulane University School of Medicine

**Keywords:** Pain Management, Pain Medicine, Team-Based Learning, TBL

## Abstract

**Introduction:**

A frequently encountered complaint in primary care, pain has a variety of causes and treatments. To reduce mortality, the Centers for Disease Control released guidelines on opioid prescribing in 2016. Taking those guidelines into account, this team-based learning (TBL) module for third-year medical students on their family medicine clinical clerkship serves as an introductory application for treatment of nociceptive and neuropathic pain.

**Methods:**

Students accessed preparticipation reading material online 1 week before the session. After students had been arranged into groups for the 1-hour module, each student completed an individual readiness assessment test (iRAT). Next, each group completed the group readiness assessment test (gRAT) using Immediate Feedback-Assessment Technique cards to give its answers. To complete the more complex learning objectives, groups answered the multiple-choice team application (tAPP) questions.

**Results:**

Over 2 years, this module was administered to more than 400 students. Average iRAT scores were approximately 80% and gRAT scores nearly 100%. Groups scored approximately 70% on the tAPP questions. Students rated the module on a 5-point Likert scale (1 = *strongly disagree,* 5 = *strongly agree*) and provided individual comments. The course always received a score of at least 4, as well as consistent positive feedback.

**Discussion:**

The module can easily be embedded into a preexisting clinical clerkship didactic series. It can also serve as a platform on which to build additional TBL modules following a more longitudinal course and more in-depth skills development in pain management.

## Educational Objectives

By the end of this module, learners will be able to:

1. Choose the most appropriate treatment regimen based on first differentiating the pathophysiology of pain and its types (i.e., nociceptive vs. neuropathic).
2. Implement the use of different classes of pain medicines using a stepwise therapeutic approach.
3. Interpret a dosage conversion chart, understanding the role of opiates in chronic pain management to discriminate alternative equipotent dosages within the same drug class.
4. Manage low back pain using the most effective and safest medication for treatment based on previous treatment, potential side effects, medication interactions, and comorbidities.

## Introduction

Family medicine is one of the most diverse medical specialties, touching on all common problems in all patient populations. Chronic pain is one of the most common reasons for seeking medical attention and is reported by 20%-50% of patients in primary care.^1^ Classification of the different types of pain is necessary to help guide treatment, which is relevant for any ambulatory care provider. Treating pain can be challenging for physicians given its different types—nociceptive and neuropathic—as well as its duration—acute and chronic—all with different treatments based on type. Additionally, there is a large psychological component to each patient's response to and experience of pain, which requires an individualized treatment approach that still adheres to safe practicing guidelines. Nociceptive pain refers to nerve activation secondary to an actual or potential tissue-damaging stimulus, while neuropathic pain results from inflammation of damaged nerves.^2^ Chronic pain is generally regarded as persistent pain lasting more than 3 months. Once the etiology of the pain has been determined, things are further complicated by the need to choose the most effective agents for its treatment, which range from procedural, psychologic, pharmacologic, and physiotherapeutic to alternative.

Given the commonality and complexity of this problem, there is a paucity of teaching resources on the topic. There are only two pain-management team-based learning (TBL) modules in *MedEdPORTAL*: “Management of Nociceptive Pain for Third-Year Medical Students”^3^ and “Minimizing the Misuse of Prescription Opioids in Patients With Chronic Non-malignant Pain.”^4^ This module covers the same topics but includes updated 2016 Centers for Disease Control (CDC) guidelines on appropriate opioid prescribing to prevent mortality,^5^ as well as the management of neuropathic pain, which previous TBL modules have not.^5^

Guidelines from the Liaison Committee on Medical Education require schools to employ both an active learning process and one that develops skills to work in team-oriented environments.^6^ TBL helps achieve both of these aims and has been shown for decades to be more effective than traditional passive lecture-based teaching. TBL is an approach that ensures students come prepared to actively participate by assigning reading material to be completed before the teaching session and having students take an individual readiness assessment test (iRAT) on that material at the beginning of the activity. Students then work in groups on the same questions, arriving at their consensus answer by taking a group readiness assessment test (gRAT). The majority of the remaining time is spent having students again work in groups on the more difficult team application (tAPP) questions, focusing on analysis rather than just on comprehension, which has been assessed by the iRAT and gRAT. Active learning approaches like TBL have been shown to produce a nearly 6% increase in exam scores in medical students.^7^ These teaching methods have also been shown to increase the success of struggling students the most, reducing failure rates by 55%.^8^

This module is a practical application of prescribing medicines to treat pain with awareness of potential side effects and drug interactions. The target audience is third-year medical students starting their first clinical rotations having only a theoretical knowledge of pharmacology. Over the past 2 years, this module has been repeatedly modified based on feedback from students, allowing for better clarification of questions and more efficient use of limited teaching time.

## Methods

### Curricular Context

Third-year medical students spent the first week of their 6-week family medicine rotation in didactics, being introduced to common topics relevant to primary care. Amongst numerous topics, they received one TBL module on continuous quality improvement/the chronic care model. The Tulane Department of Family Medicine received a Health Resources and Services Administration grant to develop a patient-centered, medical home–focused curriculum and move from a traditional lecture-based approach to an active learning, skills-driven didactic series. This process started in 2016, when the first and only TBL module administered during the third and fourth clinical years was implemented. Subsequently, the curriculum expanded in 2018 to include this particular module on pain management. Faculty training in TBL workshops aided this development. Students had been introduced to TBL during their preclinical years, amounting to 50 hours of instruction, or 5% of their lecture time.

For the family medicine clerkship, the students' grades were determined by and weighted as follows: shelf exam 50%, four timed quizzes 40%, and professionalism 10%. This module contributed to their final grade as part of the professionalism score, which required students to complete orientation, attend all lectures, and attend all clinics.

### Team Formation

The usual class size was approximately 35, and students were randomly assigned to one of six groups of no more than seven students. This was in keeping with the TBL-recommended ideal group size of five to seven.^9^ Students could be randomly assigned since they were all third-year medical students at approximately the same level of training and went through every 8-week rotation of that year in sync.

### Description of Advance Preparation Resources

Students were emailed a preparticipation reading on pain management 1 week before the teaching session (Appendix A). As per TBL teaching recommendation, the ratio of advance preparation to in-class time was approximately two to one.^9^ When surveyed, all students stated they were able to read the handout in less than 2 hours, and 1 hour was needed to complete the module.

### Description of Readiness Assurance Process

Once formed in groups, all students completed an iRAT with five multiple-choice questions covering rote learning from the reading assignment (Appendix B). Once they had individually completed the iRAT, students were instructed to wait for all other group members to finish. Thereafter, they discussed the questions and arrived at their group answers. Groups used Immediate Feedback-Assessment Technique (IF-AT) multiple-choice scratch-off cards to see if they had selected the correct answer. IF-AT forms can be ordered for a nominal cost from Epstein Educational Enterprises.^10^ Alternatively, if IT-AF forms are not on hand, a gRAT answer sheet (Appendix C) could be used: When it has been completed, the instructor could read aloud the correct answers (Appendix D). Time was allowed for students to receive immediate verbal feedback and clarifications on questions from the instructor before moving on to the tAPP (Appendix E).

### Description of tAPP Activities

Once all groups had finished the initial part of the module, they were instructed to start the tAPP, which consisted of five multiple-choice clinical vignettes. Individuals each chose their own answers; then, once all group members had done so, they discussed the questions together and came up with a consensus answer to each question. The questions involved applying the material learned to decide how to treat different patients in a clinical scenario with different medical histories, medications, and types of pain, just like the situations students would encounter on their clinical rotation. Group discussion aided answering these more complex questions, which required Bloom taxonomy's higher-level critical thinking skills of analyzing, evaluating, and creating, versus the lower-level processes of remembering and understanding, which had been assessed using the iRAT and gRAT.^11^ Once all groups had finished coming up with all their group answers, one student in each group was assigned to hold up the group's multiple-choice answer card A, B, C, or D (Appendix F). This directly followed the TBL tAPP's 4S principles of significant problem, same problem, specific choice, and simultaneous reporting.^6^ The instructor went question by question, first reading a question aloud, then having the groups simultaneously hold up their group answers. The instructor went group by group, asking for the rationale for an answer; once all groups had given their explanations, the correct answer was revealed (Appendix G). Time was allowed for students to ask questions regarding the explanation, and once completed, the process was repeated, reading the next question and having simultaneous group reporting. Time was given at the end of class, once the tAPP was done, for appeals (Appendix H). The instructor asked if the students had any difficulty understanding any of the concepts in the preparticipation reading and if they felt that it adequately prepared them to answer the questions. The class was then allowed time to appeal both the iRAT and tAPP questions either in writing or verbally. Lastly, time for comments regarding the overall format and usefulness of the TBL was allowed.

### Facilitation Schema

The instructor designed this single module to be completed in 60 minutes. A suggested facilitation time line is given below:

• Welcome and learning objectives (5 minutes).
• iRAT (5 minutes).
• gRAT (10 minutes).
• Time allowed for clarification of difficult questions (5 minutes).
• tAPP (30 minutes).
• Appeals (5 minutes).

### Evaluation

For each teaching session at Tulane, students completed an online evaluation using SurveyMonkey right after the lesson. Assessment of students' learning was achieved though iRAT, gRAT, and tAPP scores.

## Results

The family medicine clerkship didactics series has implemented this module for the past 2 years. Up to this point, over 400 mainly third-year medical students have completed the module. For each teaching session, students were required to complete an online survey evaluating the course. The results were routinely reviewed, and comments were used to further improve students' learning. Students consistently achieved at least 80% correct answers on the iRAT and nearly 100% on the gRAT. These results, compared with other TBL modules, demonstrated an average improvement in scores of at least 14% when working as a group versus as an individual.^12^

On the more challenging tAPP questions, students on average scored 70%. The online survey allowed students to rate the overall clinical rotation, as well as each teaching session, using a 5-point Likert scale (1 = *strongly disagree,* 5 = *strongly agree*) and to provide individual comments. Due to this module being stand-alone rather than part of a series, peer evaluations were not included. The rotation and individual courses all consistently obtained an average score of at least 4. Some comments were as follows:

• “The reading was informative and very helpful.”
• “This session was helpful in tying in what we learned from the reading.”
• “Good to learn alongside peers.”
• “Good topic we don't learn about anywhere else.”
• “I think it was useful to have a session about chronic pain as it is an important primary care topic.”

## Discussion

The student comments from the surveys over the past 2 years indicate that the goal of having an interactive practical skills-based introduction to pain management in a group setting was achieved. Survey data helped to further improve the learning experience. The 6-week family medicine clerkship is relatively short compared to the other required clerkships, which are all 8 weeks and, given the diverse nature of the specialty, must cover a wide range of topics. Over the past 4 years, the curriculum has changed from traditional lectures to an active, skills-based learning approach incorporating important orphan topics, such as the other TBL module on continuous quality improvement/the chronic care model as well as this one on pain management. This module is meant to be a valuable first-time practical skills-based application of pharmacology in the setting of pain management for medical students starting their family medicine clinical rotation. It is unique among TBL modules as it addresses the 2016 CDC guidelines regarding opioid prescribing in light of the current epidemic and covers nociceptive as well as neuropathic pain treatment.

Two particular lessons were learned that, once focused on, helped to better manage time in the session: Giving groups a time limit for each section kept the tAPP part of the module in particular from going overlong. Students' survey comments that time was inefficiently used during this part of the exercise because different groups completed it at greatly different times inspired this change. Also, calculating morphine-equivalent dosages took groups the greatest amount of time. The tAPP material was updated to include the conversion chart from the preparticipation material to better guide students. Thereafter, groups spent much less time on the question and needed little clarification during the session.

From the facilitator standpoint, TBL can also occur remotely in real time. Along with all other nonclinical clerkship teaching at the university, this module had to be adapted for remote learning in March 2020 to allow for social distancing. This was achieved using Zoom: Each class was sent an online invitation along with instructions and preparticipation reading. At the beginning of class, students were placed in groups by being randomly assigned to breakout rooms, which is a feature of the platform. The facilitator hosting the meeting was able to open and close the rooms when needed for both individual group work and entire-class reporting and discussion. Four alterations to the traditional TBL format were made as a result of the module not being in person.

First, it was not possible to use IT-AF cards, so when the gRATs were complete the rooms were closed and each group reported its answers. Once all groups had done so, they explained their reason for choosing their answer following the tAPP reporting process. The correct answers were then given along with their explanations. Second, the change in format slowed down the process of transitioning from individual to group to entire-class work, as well as the reporting of answers. Time allowed for the module was therefore increased from 60 to 90 minutes, which proved adequate. Third, during the gRAT and tAPP, the facilitator joined each individual group briefly to see if there were any technical difficulties. Fourth, the tAPP answer cards could not be used, so there was no simultaneous reporting. To compensate for this, the facilitator went to each group and asked for its answer, which resulted in rapid reporting, before having groups give the rationale for the answers they chose. Assistance was given to the facilitator on how to host a Zoom meeting generally, as well as how to create, join, open, and close breakout rooms. This proved relatively easy. Technical support was available but not needed.

One limitation of this module is that whether it has resulted in an improvement in standardized test scores is not known. In the future, scores on shelf exam questions covering pain management could be compared with scores from students who took the exam prior to the inclusion of this module or by not offering this module on every block and comparing test scores between blocks. Another limitation of the module is that it is meant to be an introduction to the topic and does not provide in-depth coverage of other treatment modalities for managing chronic pain, such as physical therapeutic, psychological, and surgical approaches. It is also a stand-alone module, given the limited time available with each group of clerkship students, and does not allow for multiple sessions or peer evaluations, which are part of traditional TBL grading. However, given its short length, the module can easily be embedded into a preexisting didactic series wishing to cover this topic.

A future direction is to expand the number of sessions using the current module as the introduction and to cover more nonpharmacologic treatments, as well as diagnostic evaluations. This could be achieved by teaching in a setting where more time can be had with learners, such as with our institution's affiliated family medicine residency program, which has prolonged regular clinical and nonclinical teaching sessions.

## Appendices

Pain Management TBL Advance Preparation Resources.docxPain Management TBL iRAT.docxPain Management TBL gRAT Group Answer Form.docxPain Management TBL gRAT Answer Key.docxPain Management TBL Team Application.docxPain Management TBL Team Application Answer Cards.docxPain Management TBL Team Application Answer Key.docxPain Management TBL Appeals Form.docx
All appendices are peer reviewed as integral parts of the Original Publication.
