# Supplementary material for: Introduction to Pain Management for Third-Year Medical Students Team-Based Learning Module
Source: MedEdPORTAL. 2021 Feb 11;17:11095. doi: 10.15766/mep_2374-8265.11095 (PMC7880255; doi:10.15766/mep_2374-8265.11095)
Supplement: Supplementary file 1 — Pain Management TBL Advance Preparation Resources.docxPain Management TBL iRAT.docxPain Management TBL gRAT Group Answer Form.docxPain Management TBL gRAT Answer Key.docxPain Management TBL Team Application.docxPain Management TBL Team Application Answer Cards.docxPain Management TBL Team Application Answer Key.docxPain Management TBL Appeals Form.docx [file mep_2374-8265.11095-s001.zip › A. Pain Management TBL Advance Preparation Resources.docx]

Team Based Learning Pain Management Module

Educational Objectives

By the end of this session, learners will be able to:

1. Choose most appropriate treatment regimen based on first differentiating the pathophysiology of pain and its types, i.e. nociceptive vs. neuropathic

2. Implement the use of different classes of pain medicines using a step-wise therapeutic approach

3. Interpret a dosage conversion chart understanding the role of opiates in chronic pain management to discriminate alternative equipotent dosages within the same drug class

4. Manage low back pain implementing the most effective and safest medication for treatment based on previous treatment, potential side-effects, medication interactions and co-morbidities

PRE-READING MATERIALS

Chronic pain is among the most common reasons for seeking medical attention and is reported by 20 to 50% of patients in primary care. Patients with chronic pain require ongoing evaluation, education and reassurance, as well as help in setting reasonable expectations for response. Currently available treatment modalities on average result in only about 30% decrease in pain. But even a partial response of 30% can be clinically significant and improve a patient’s quality of life.^1^

To guide patient-specific selection of therapy, clinicians should evaluate patients and establish or confirm the diagnosis. Detailed recommendations on diagnosis are provided in other guidelines but evaluation should generally include a focused history, including history and characteristics of pain and potentially contributing factors (e.g., function, psychosocial stressors, sleep) and physical exam, with imaging or other diagnostic testing only if indicated (e.g., if severe or progressive neurologic deficits are present or if serious underlying conditions are suspected).^2^ For complex pain syndromes, pain specialty consultation can be considered to assist with diagnosis as well as management. Diagnosis can help identify disease-specific interventions to reverse or ameliorate pain; for example, improving glucose control to prevent progression of diabetic neuropathy; immune-modulating agents for rheumatoid arthritis; physical or occupational therapy to address posture, muscle weakness, or repetitive occupational motions that contribute to musculoskeletal pain; or surgical intervention to relieve mechanical/compressive pain.^2^

The underlying mechanism for most pain syndromes can be categorized as neuropathic (e.g., diabetic neuropathy, postherpetic neuralgia, fibromyalgia), or nociceptive (e.g., musculoskeletal conditions, inflammation, mechanical/compressive problems). Neuropathic pain, resulting from damage to or pathology within the central nervous system, can be central or peripheral. Nociceptive pain, in contrast, is caused by stimuli that threaten or provoke actual tissue damage. The diagnosis and pathophysiologic mechanism of pain have implications for symptomatic pain treatment with medication. For example, evidence is limited or insufficient for improved pain or function with long-term use of opioids for several chronic pain conditions for which opioids are commonly prescribed, such as low back pain, headache, and fibromyalgia.^2^ Although NSAIDs can be used for exacerbations of nociceptive pain, other medications (e.g., tricyclics, selected anticonvulsants, or transdermal lidocaine) generally are recommended for neuropathic pain. In addition, improvement of neuropathic pain can begin weeks or longer after symptomatic treatment is initiated.^2^

Medications should be used only after assessment and determination that expected benefits outweigh risks given patient-specific factors. For example, clinicians should consider fall risk when selecting and dosing potentially sedating medications such as tricyclics, anticonvulsants, or opioids, and should weigh risks and benefits of use, dose, and duration of NSAIDs when treating older adults as well as patients with hypertension, renal insufficiency, or heart failure, or those with risk for peptic ulcer disease or cardiovascular disease. Some guidelines recommend topical NSAIDs for localized osteoarthritis (e.g., knee osteoarthritis) over oral NSAIDs in patients aged ≥ 75 years to minimize systemic effects.^2^

The main consensus is that the majority of patients can be treated with acetaminophen or nonsteroidal anti-inflammatory drugs (NSAIDs). The guideline below contains 14 recommendations regarding the long-term use of opioids, to include such areas as initiating and titrating opioid therapy, methadone use, monitoring opioid use, opioid side effects, and opioid management plans. Primary care physicians may find this guideline helpful if it is necessary to prescribe opioids in the treatment of chronic pain.

| American Pain Society and American Academy of Pain Medicine treatment guidelines for noncancer pain recommendations for clinicians |
| --- |
| 1. Select patient for chronic opioid use using risk stratification 2. Communicate risks and benefits of chronic opioid use and potential use of pain management plan for documentation with patient 3. Consider initial use of opioid therapy on a trial basis, with individualized drug choice, initial dosing and titration 4. Use caution if methadone is selected, and use caution during initiation and titration by clinicians familiar with its unique properties 5. Perform monitoring for efficacy, adverse effects, and possible diversion 6. Frequent monitoring and possible consultation with behavioral health specialist for patients with history of drug abuse or psychiatric issues 7. Reassess for benefits and risks of opioid use and consider opioid rotation if patient receiving inadequate efficacy or intolerable adverse effects while attempting to escalate opioid dosage 8. Anticipate and treat opioid-related adverse effects 9. Integrate psychotherapeutic interventions, interdisciplinary therapy, and adjunctive nonopioid therapies 10. Counsel patients on potential for cognitive impairment that may affect daily activities (ie, driving) 11. Help patients identify a medical home responsible for their overall care 12. Consider as needed opioids for breakthrough pain if opioids are prescribed around the clock 13. Counsel female patients about the benefits and risks of chronic opioid therapy during pregnancy and after delivery 14. Familiarize themselves with state and federal regulations regarding opioid prescribing |

GENERAL TREATMENT PRINCIPLES FOR LOW BACK PAIN

In general, the treatment of acute and chronic low back pain involves a balance between patient expectations for pain relief and the amount of pain relief that medications can provide.^2^ Early patient education regarding what can be expected from pharmacotherapy will lead to more realistic expectations for the patient. Psychosocial factors and emotional distress are stronger predictors of outcomes of low back pain than either physical examination findings or severity and duration of pain. Physicians should assess patients for depression, unemployment, job dissatisfaction, somatization disorder, and psychological distress, as these conditions tend to delay recovery.^2^

When deciding to prescribe medications for the treatment of low back pain, the clinician should weigh the benefits against the risks of the medications as they relate to the specific patient to be treated. By and large, medication use for low back pain should be for the shortest time possible and discontinued when there appears to be no more benefit to its use.^2^ Extended courses of medications should only be used if the patient clearly exhibits continued benefits from therapy without major adverse effects. Throughout the treatment period, documentation of patient’s pain relief and quality of life subsequent to prescribed therapies is crucial. Pain scores should be documented in the patient’s record as the fifth vital sign, using a visual analog scale, or on a scale of 1 up to a maximum of 10.

Published guidelines for the treatment of low back pain recommend that clinicians select medications with documented efficacy data and use these in combination with education and self-care in the treatment plan for a patient with low back pain.^2^ Before prescribing any medications for low back pain, the guidelines recommend that clinicians complete a variety of patient assessments, to include pain and functional deficits and a benefits/risk analysis of the medications, in an attempt to select the best care for the patient.^2^ In addition, costs of medications must be considered as part of the treatment plan.

At best, the medications used to treat low back pain have been shown to be moderately effective for short-term treatment of acute back pain. With medication, thankfully most patients (90%) recover within a couple of weeks of the onset of low back pain.^2^ For some patients it can take up to 2 months or more for recovery to occur.^2^ Unfortunately, some patients continue to have intermittent or chronic low back pain for 6 months or more.^2^ Social, clinical, and economic factors may help predict which patients may transition from acute to chronic low back pain.^2^

MEDICATIONS FOR THE TREATMENT OF LOW BACK PAIN

Acetaminophen

An Australian trial, randomly allocated patients with acute low-back pain in a 1:1:1 ratio to receive up to 4 weeks of regular doses of paracetamol (three times per day; equivalent to 3990 mg paracetamol per day), as-needed doses of paracetamol (taken when needed for pain relief; maximum 4000 mg paracetamol per day), or placebo. Findings suggest that regular or as-needed dosing with paracetamol does not affect recovery time compared with placebo in low-back pain, and question the universal endorsement of paracetamol in this patient group.^3^

Even without evidence to support its use, many consider acetaminophen to be the drug of choice to alleviate low back pain.^2^ Acetaminophen is a reasonable choice, because of its more favorable safety profile and low cost compared with other treatment options.

In 2011, the Food and Drug Administration (FDA) dictated limits regarding the maximum tablet strength (325 mg) for prescription acetaminophen-containing products (ie, Vicodin, Lortab, and so forth).^2^ The limit on tablet strength allows providers to continue to prescribe 1 to 2 tablets per day every 4 hours and keep the patient’s daily acetaminophen intake under the 4000 mg maximum daily intake level. Over- the-counter acetaminophen-containing products are not affected by this requirement.

When using acetaminophen for treatment of low back pain, dosing should start at the maximal recommended doses. A typical dosing regimen for acetaminophen would be 1000 mg orally every 6 hours, with a maximum daily dose of 4000 mg.^2^ Maximum doses should be used for the shortest time possible to minimize occurrence of adverse events. The adverse effect of greatest concern with acetaminophen is liver toxicity. Data from surveillance programs show that in an 8-year period (1990–1998) there were around 56,000 visits to emergency rooms, 26,000 hospitalizations, and 458 deaths due to acetaminophen overdoses.^2^ Consumption of alcohol while using acetaminophen containing products increases the risk of liver toxicity. Patients who consume alcohol or have concurrent liver toxicity need to limit their daily acetaminophen intake to 2000 mg.

Nonsteroidal Anti-Inflammatory Drugs

NSAIDs are another option for the treatment of low back pain. Research supports the use of NSAIDs over placebo as treatment for acute back pain.^2^ For chronic pain, the data are less convincing. In comparing NSAIDs with acetaminophen, NSAIDs have proved to be more effective but with more risks attached.^2^ Clinicians should consider NSAIDs for the treatment of low back pain if a patient fails to respond to acetaminophen after an appropriate trial period.^2^

Clinicians today can choose from a wide variety of different NSAIDs on the market to treat low back pain.^2^ If a drug from one class does not provide relief, another agent from a different class could be tested for efficacy in the patient. Similar to acetaminophen, maximal recommended doses should be initially prescribed. The use of aspirin cannot be recommended because there are insufficient data to support or refute its use as a treatment for low back pain.^2^ No evidence exists that cyclooxygenase-2 inhibitors (celecoxib/Celebrex) for low back pain treatment provides any additional efficacy compared with traditional NSAIDs.^2^

NSAIDs commonly used to treat low back pain

Class/Drug Oral Dosing

Propionic Acid Derivatives

Ibuprofen 200–400 mg every 4–6 h (maximum dose 3200 mg)

Naproxen 250mg 3to 4 times daily

Acetic Acid Derivatives

Sulindac 150–200 mg twice daily

Etodolac 200–400 mg 3 to 4 times daily (maximum 1000 mg/d)

Ketorolac 10 mg every 4–6 h (maximum 40 mg/d, no more than 5 d)

Diclofenac 50 mg 3 times daily (maximum 150 mg)

Enolic Acid Derivatives

Piroxicam 10–20 mg daily to twice daily (maximum 20 mg)

Meloxicam 7.5–15 mg/d (maximum 15 mg)

Nabumetone 500–1000 mg daily to twice daily (maximum 2000 mg)

Cyclooxygenase-2 Selective Inhibitors

Celecoxib 100–200 mg daily or twice daily

Safety issues, mainly renal and gastrointestinal toxicities, involving the use of NSAIDs must be considered when deciding to prescribe this class of drugs for low back pain. NSAID-induced acute renal failure ranks second only to aminoglycoside- induced acute renal failure.^2^ NSAID-induced acute renal failure accounts for about 15% of all drug-induced renal failure. Nephrotoxicity occurs mainly as a result of prostaglandin inhibition and is usually, but not always, reversible.^2^ Interstitial nephritis can occur, which can lead to permanent kidney damage.2 Gastrointestinal toxicity, also resulting from prostaglandin inhibition, occurs at a rate of 1% to 2% of all NSAID use.^2^ Cyclooxygenase-2 inhibitors may have fewer gastrointestinal toxicities in comparison with traditional NSAIDs.^2^

If a traditional NSAID is selected for treatment, strategies to prevent NSAID-induced gastrointestinal toxicities include combining NSAID therapy with misoprostol, H2-receptor blockers, or proton-pump inhibitors.^2^ In addition, concern exists for the use of NSAIDs and subsequent cardiovascular toxicity, mainly myocardial infarction. According to the literature, the risk appears to be increased over that of nonusers.^2^ In general, for patients at increased risk of cardiovascular toxicities, ibuprofen, diclofenac, and meloxicam should be avoided.^2^ Naproxen is the drug of choice, as it appears that it does not increase the risk of cardiovascular events.^2^ Because of potential renal, gastrointestinal, and cardiovascular concerns, NSAIDs should be used for the shortest treatment period possible to potentially minimize these concerns.

Tramadol

Only after the patient has failed acetaminophen and NSAID therapy should tramadol be considered an option for the treatment of low back pain.^2^ Tramadol exhibits weak opioid effects and inhibits serotonin and norepinephrine reuptake. Short-term improvements in pain and function have been demonstrated for tramadol (dosed at 50 mg, maximum 400 mg per day for 4 weeks) in the treatment of low back pain, but there is a lack of long-term data.^2^ Two studies report that tramadol (37.5 mg) in combination with acetaminophen (325 mg) report improvements in pain in two 3-month trials.^2^

Major side effects, occurring at a rate greater than 10%, associated with tramadol include dizziness, nausea, sedation, constipation, and headache.^2^ Tramadol use at or above recommended doses has also been associated with seizures.^2^ Patients receiving serotonin reuptake inhibitors (SSRIs), tricyclic antidepressants, monoamine oxidase inhibitors, neuroleptics, or other opioids have an even higher potential for occurrence of seizures.^2^ Tramadol has a large number of drug interactions, many involving the cytochrome P450 enzyme system (2B6, 2D6, and 3A4).^2^

If a clinician is considering prescribing tramadol for a patient with low back pain, it would be prudent to perform a complete review of medication to avoid the possibility of drug interactions. Tramadol use should be avoided in patients with a history of opioid addiction, as reinitiation of physical dependence to opioids can occur.^2^ Clinicians should be alert to signs of dependence and abuse among patients using tramadol, even if there is no prior history of opioid dependence.

Opioids

Long-term use of opioids in the treatment of low back pain is not recommended.^2^ A Cochrane review of opioids for chronic low back pain only identified one trial, in which opioids were compared with naproxen.^2^ Evidence showed that opioids provided greater pain relief and improve mood better than NSAIDs, but patient activity levels were not improved. Opioids exhibit a variety of adverse effects, mainly central nervous system related (drowsiness, dizziness, confusion), cardiovascular (bradycardia, hypotension), and gastrointestinal (constipation and nausea).^2^ In addition, they have a high potential for abuse, misuse, and addiction. Regarding populations potentially at greater risk for harm, risk is greater for patients with sleep apnea or other causes of sleep-disordered breathing, patients with renal or hepatic insufficiency, older adults, pregnant women, patients with depression or other mental health conditions, and patients with alcohol or other substance use disorders. Interpretation of clinical data on the effects of opioids on sleep-disordered breathing is difficult because of the types of study designs and methods employed, and there is no clear consensus regarding association with risk for developing obstructive sleep apnea syndrome.^4^ However, opioid therapy can decrease respiratory drive, a high percentage of patients on long-term opioid therapy have been reported to have an abnormal apnea-hypopnea index, opioid therapy can worsen central sleep apnea in obstructive sleep apnea patients, and it can cause further desaturation in obstructive sleep apnea patients not on continuous positive airway pressure (CPAP).4 Reduced renal or hepatic function can result in greater peak effect and longer duration of action and reduce the dose at which respiratory depression and overdose occurs.^4^ Age-related changes in patients aged ≥65 years, such as reduced renal function and medication clearance, even in the absence of renal disease, result in a smaller therapeutic window between safe dosages and dosages associated with respiratory depression and overdose. Older adults might also be at increased risk for falls and fractures related to opioids.^4^

  When starting opioid therapy for chronic pain, clinicians should prescribe immediate-release opioids instead of extended-release/long-acting (ER/LA) opioids (recommendation category: A, evidence type: 4).^4^ ER/LA opioids include methadone, transdermal fentanyl, and extended-release versions of opioids such as oxycodone, oxymorphone, hydrocodone, and morphine. The clinical evidence review found a fair-quality study showing a higher risk for overdose among patients initiating treatment with ER/LA opioids than among those initiating treatment with immediate-release opioids.^4^ The clinical evidence review did not find evidence that continuous, time-scheduled use of ER/LA opioids is more effective or safer than intermittent use of immediate-release opioids or that time-scheduled use of ER/LA opioids reduces risks for opioid misuse or addiction.^4^

In 2014, the FDA modified the labeling for ER/LA opioid pain medications, noting serious risks and recommending that ER/LA opioids be reserved for “management of pain severe enough to require daily, around-the-clock, long-term opioid treatment” when “alternative treatment options (e.g., nonopioid analgesics or immediate-release opioids) are ineffective, not tolerated, or would be otherwise inadequate to provide sufficient management of pain” and not used as “as needed” pain relievers.^4^ FDA has also noted that some ER/LA opioids are only appropriate for opioid-tolerant patients, defined as patients who have received certain dosages of opioids (e.g., 60 mg daily of oral morphine, 30 mg daily of oral oxycodone, or equianalgesic dosages of other opioids) for at least 1 week.^4^ Time-scheduled opioid use can be associated with greater total average daily opioid dosage compared with intermittent, as-needed opioid use (contextual evidence review). In addition, experts indicated that there was not enough evidence to determine the safety of using immediate-release opioids for breakthrough pain when ER/LA opioids are used for chronic pain outside of active cancer pain, palliative care, or end-of-life care, and that this practice might be associated with dose escalation.

Instead of a first-line therapy for low back pain, opioids are more appropriately prescribed as either second-line or third-line therapy for severe, acute back pain.^2^ Patients who have significantly reduced quality of life owing to their low back pain, or those with nonsurgical pathology for their back pain, may benefit from opioid treatment.^2^

If opioids are selected as part of the treatment plan for a patient with low back pain, it would be prudent to follow the World Health Organization stepwise pain treatment algorithm, starting with weak opioid/acetaminophen combination product and progressing to a strong opioid if the patient fails an adequate trial of the weak opioid.^2^ The selection of which opioid to use should be determined by obtaining a thorough patient history to elicit past opioid use and response to therapy, coexisting health disorders, allergies, concurrent drugs, and financial constraints.

Transitioning among different opioids is possible, and current recommendations for equipotent dosing are given below.^2^ If a patient cannot tolerate one opioid, a different opioid can be selected. Although there is no ceiling dose for opioids, the guidelines for the use of opioid therapy for chronic pain suggest that if a maximum dose of 120 to 180 mg of morphine (or equivalent) is reached, the patient should be referred to a pain management clinic before further escalating the opioid dose.^2^

When prescribing chronic opioid therapy, the clinician should require the patient to sign a pain contract or opioid agreement that defines the rules patients must follow to take these drugs safely. Common requirements of pain contracts include submitting to random urine and blood drug tests, using only one pharmacy to obtain medications, and obtaining prescriptions from only one provider. Contracts often state that if these requirements are not followed, the physician reserves the right to terminate care of the patient. The use of pain contracts is spurred by recent issues with opioid diversion.

Regarding high-dose therapy, several epidemiologic studies that were excluded from the clinical evidence review because patient samples were not restricted to patients with chronic pain also examined the association between opioid dosage and overdose risk.^4^ Consistent with the clinical evidence review, the contextual review found that opioid-related overdose risk is dose-dependent, with higher opioid dosages associated with increased overdose risk. Two of these studies as well as the two studies in the clinical evidence review, evaluated similar MME (Morphine Milligram Equivalent)/day dose ranges for association with overdose risk. In these four studies, compared with opioids prescribed at <20 MME/day, the odds of overdose among patients prescribed opioids for chronic nonmalignant pain were between 1.3 and 1.9 for dosages of 20 to <50 MME/day, between 1.9 and 4.6 for dosages of 50 to <100 MME/day, and between 2.0 and 8.9 for dosages of ≥100 MME/day. Compared with dosages of 1-<20 MME/day, absolute risk difference approximation for 50-<100 MME/day was 0.15% for fatal overdose and 1.40% for any overdose and for ≥100 MME/day was 0.25% for fatal overdose and 4.04% for any overdose.^4^

A study of Veterans Health Administration patients with chronic pain found that patients who died of overdoses related to opioids were prescribed higher opioid dosages (mean: 98 MME/day; median: 60 MME/day) than controls (mean: 48 MME/day, median: 25 MME/day).^4^ Finally, another recent study of overdose deaths among state residents with and without opioid prescriptions revealed that prescription opioid-related overdose mortality rates rose rapidly up to prescribed doses of 200 MME/day, after which the mortality rates continued to increase but grew more gradually.^4^

Methadone is not recommended unless the provider is experienced in its use. Its long half-life carries the potential for progressive medication accumulation and risk of overdose and death days to weeks after initiating dosing. It has many drug interactions and increases the risk of sedation and respiratory depression when combined with other CNS depressants as well as interactions at the P450 3A4 and 2D6 enzymes, potentially causing changes in blood levels of methadone and/or other prescribed medications. There was found to be a 700 percent increase in overdose/poisoning deaths associated with methadone between 1998 and 2006 (Eckholm 2008) and the FDA issued a “black box” warning in November 2006 addressing risk of overdose and QT prolongation.

Commonly Used Long‐Acting Opioids

| Compound | Strengths | Typical Starting Dose and Dosing Interval | Name Branding |
| --- | --- | --- | --- |
| Morphine controlled release tablet | 15, 30, 60, 100, 200 mg | 15‐30 mg every 8‐12 hours | MS Contin® Oramorph® SR |
| Morphine controlled‐ release capsule | 20, 30, 50, 60, 100 mg | 20 mg every 12 or 24 hours | Kadian® |
| Morphine extended‐release capsule | 30, 60, 90, 120 mg | 30 mg every day | Avinza® |
| Oxycodone controlled‐ release | 10, 20, 40, 80 mg | 10 mg every 12 hours | OxyContin® |
| Oxymorphone extended‐ release | 5, 10, 20, 30, 40 mg | 5 mg every 12 hours | Opana® ER |
| Hydromorphone extended‐ release | 8, 12, 16 mg | 8 mg once daily | Exalgo® ER |
| Fentanyl transdermal | 25, 50, 75, 100 microgram/hr patch | 25 mcg applied every 3 days | Duragesic® |
| Buprenorphine transdermal | 5, 10, 20 microgram/hr patch | 5 mcg applied every 7 days | Butrans® |

*^2^*

| **Equipotent Doses of Opioid Analgesics** |  |  |
| --- | --- | --- |
| **Oral/Rectal Dose (mg)** | **Analgesic** | **Parenteral Dose (mg)** |
| 100 | Codeine | 60 |
| - | Fentanyl | 0.1 |
| 15 | Hydrocodone | - |
| 4 | Hydromorphone | 1.5 |
| 2 | Levorphanol | 1 |
| 150 | Meperidine | 50 |
| 15 | Morphine | 5 |
| 10 | Oxycodone | - |

^2^

Muscle Relaxants

Two categories of muscle relaxants exist. Antispastic agents (baclofen, tizanidine, dantrolene, and diazepam) carry an indication for spasticity related to injury to the central nervous system (ie, multiple sclerosis) and are not recommended for treatment of low back pain.^2^ The other category, antispasmodic agents (cyclobenzaprine, methocarbamol, carisoprodol, metaxalone) can be added to treatment plans for low back pain if patients do not respond adequately to first-line analgesics.^2^ These agents should be used for the shortest time possible, preferably for no more than 2 weeks in total. Compared with placebo, muscle relaxants show better efficacy in reducing pain and relieving symptom but are less effective than NSAIDs, and patients who use muscle relaxants incur the possibility of more side effects from this class of drugs.^2^

The major side effects associated with muscle relaxants are related to the central nervous system, mainly sedation and dizziness. Benzodiazepines are not recommended to be used for chronic low back pain.^2^ These agents portray similar efficacy to skeletal muscle relaxants for acute low back pain, but should not be used for more than 3 or 4 weeks in total. Risks for abuse, addiction, and tolerance outweigh the potential benefit of benzodiazepines for the treatment of low back pain.

Clinicians should avoid prescribing opioid pain medication and benzodiazepines concurrently whenever possible (recommendation category: A, evidence type: 3).^4^ Benzodiazepines and opioids both cause central nervous system depression and can decrease respiratory drive. Concurrent benzodiazepine prescription with opioid prescription was found to be associated with a near quadrupling of risk for overdose death compared with opioid prescription alone.^4^ Experts agreed that although there are circumstances when it might be appropriate to prescribe opioids to a patient receiving benzodiazepines (e.g., severe acute pain in a patient taking long-term, stable low-dose benzodiazepine therapy), clinicians should avoid prescribing opioids and benzodiazepines concurrently whenever possible. In addition, given that other central nervous system depressants (e.g., muscle relaxants, hypnotics) can potentiate central nervous system depression associated with opioids, clinicians should consider whether benefits outweigh risks of concurrent use of these drugs.

Clinicians should check the Prescription Drug Monitoring Program (PDMP) for concurrent controlled medications prescribed by other clinicians and should consider involving pharmacists and pain specialists as part of the management team when opioids are co-prescribed with other central nervous system depressants. Because of greater risks of benzodiazepine withdrawal relative to opioid withdrawal, and because tapering opioids can be associated with anxiety, when patients receiving both benzodiazepines and opioids require tapering to reduce risk for fatal respiratory depression, it might be safer and more practical to taper opioids first. Clinicians should taper benzodiazepines gradually if discontinued because abrupt withdrawal can be associated with rebound anxiety, hallucinations, seizures, delirium tremens, and, in rare cases, death (contextual evidence review). A commonly used tapering schedule that has been used safely and with moderate success is a reduction of the benzodiazepine dose by 25% every 1–2 weeks.^4^

Cognitive behavioral therapy (CBT) is a counseling method in which patients are taught to identify their negative thinking patterns and change them as well as their subsequent unhealthy behaviors. It has been shown to increase tapering success rates and might be particularly helpful for patients struggling with a benzodiazepine taper.^4^ If benzodiazepines prescribed for anxiety are tapered or discontinued, or if patients receiving opioids require treatment for anxiety, evidence-based psychotherapies (e.g., CBT) and/or specific anti-depressants or other nonbenzodiazepine medications approved for anxiety should be offered. Experts emphasized that clinicians should communicate with mental health professionals managing the patient to discuss the patient’s needs, prioritize patient goals, weigh risks of concurrent benzodiazepine and opioid exposure, and coordinate care.

The different skeletal muscle relaxants are not pharmacologically related, therefore various drugs may exhibit different efficacy and safety profiles in a patient. The major metabolite of carisoprodol is meprobamate, which exhibits abuse and overdose issues.^2^ There is a black-box warning associated with dantrolene use for potentially fatal hepatotoxicity, and tizanidine has reports of reversible hepatotoxicity associated with its use.^2^ Selection of which muscle relaxant to use should be based on adverse effects, drug interactions, and cost. Three of the muscle relaxants (carisoprodol, meprobamate, and cyclobenzaprine) are listed on the Beers list of inappropriate medications for older adults (65 years).^2^ Of the muscle relaxants available, the most research exists for cyclobenzaprine and tizanidine.^2^ If the decision is made to use a muscle relaxant for treatment of low back pain, cyclobenzaprine should be considered first, because of the risk of hepatotoxicity with tizanidine use.

Antidepressants

Antidepressants may be prescribed for the treatment of chronic low back pain, but are not an appropriate choice for acute low back pain.^2^ Antidepressants are effective in treating neuropathic pain with a number needed to treat of 2 to 3 for 1 patient to achieve a 50% reduction in pain.^1^ Tricyclic antidepressants and serotonin norepinephrine reuptake inhibitors possess analgesic qualities while the evidence for the effectiveness of selective serotonin reuptake inhibitors is weaker.^1^ The mechanism of action is unclear since TCAs with the greatest effect on serotonin are the most effective while fluoxetine, a potent serotonin reuptake inhibitor has little to no effect on reducing pain.

While effective at treating pain, TCAs have various side effects depending on the agent. Side effects include anticholinergic, antihistaminergic (doxepin has the most antihistaminergic effects) and alpha-1 adrenergic receptor blockade, and cardiac effects (increasing intraventricular conduction, prolonged QT interval, and prolonged AV nodal conduction). Amitriptyline has the most potent anticholinergic effects and despiramine has the least and is thus the least sedating. Anticholinergic effects include dry mouth, orthostatic hypotension, constipation, and urinary retention. Randomized clinical trials demonstrate that tricyclic antidepressants (amitriptyline, nortriptyline, desipramine), which exhibit some norepinephrine reuptake inhibition, offer some pain reduction in patients without clinical depression, but no studies have been done in patients who suffer from comorbid depression.^2^ Functional improvement did not occur, and greater than 20% of the patients had side effects. Antidepressants with both norepinephrine and serotonin reuptake inhibition, namely duloxetine, venlafaxine, and bupropion, have achieved pain reduction for certain conditions (ie, peripheral neuropathy). A systematic review of 6 RCTs (3 in patients with diabetic neuropathy and 3 with fibromyalgia) found evidence that duloxetine 60mg daily was more effective than placebo with response rates similar to other antidepressants, but there are no direct comparison trials available.^1^

Antidepressant medications may be helpful in treating patients with low back pain who also suffer from depression.^2^ Upward of 50% of patients with chronic low back pain exhibit depressive symptoms. It is essential that the treatment plan for low back pain include the treatment of depression, if present, as patients who receive treatment for both conditions have better outcomes. Analgesic antidepressants may provide pain relief separate from the antidepressant effects since analgesic effects appear to occur earlier (~ 1 wk) and at lower doses than for antidepressant effects. In addition, the analgesic effect of antidepressants in neuropathic pain has been established in nondepressed patients.

Antipsychotic Drugs

Preclinical studies in humans theorize that excessive dopamine may be linked with pain syndromes.^2^ It would therefore be reasonable to investigate antipsychotics as an option for pain management. A Cochrane review examined the use of anti- psychotics for acute and chronic pain in adults.^2^ The purpose of the review was to examine efficacy and adverse effects of this drug class in acute or chronic pain. Eleven studies were identified and reviewed, but none of them involved patients with acute or chronic low back pain specifically. Six studies focused on chronic pain management, mainly for cancer pain, headaches, and neuralgic pain. The evidence did not strongly support the use of antipsychotics for chronic pain treatment, even when the trials were combined in a meta-analysis. Many of these trials used first-generation antipsychotics (ie, fluphenazine, thioridazine, prochlorperazine, haloperidol), and none investigated second-generation agents.

Because of the risks and adverse effects associated with antipsychotic drugs, the potential problems with this drug class outweigh the benefits of their use. Adverse effects seen with first-generation antipsychotics include cardiovascular events (QT prolongation, arrhythmias, and so forth), central nervous system events (dystonic reactions, akathisia, extrapyramidal reactions, and so forth), and endocrine events (glucose irregularities and sexual dysfunction). Without confirmed evidence showing efficacy for treatment of low back pain, antipsychotics should not be recommended as therapy. Research involving second-generation antipsychotics may provide evidence for their use, but at present there is none.

Antiseizure Medications

Gabapentin and carbamazepine, both anticonvulsants, have been used to treat chronic low back pain and have been proved to be efficacious for the treatment of sciatica.^4^ Gabapentin has a high affinity for voltage-gated calcium channels, which may modulate the release of excitatory neurotransmitters that affect nociception.^2^ Gabapentin has primarily been studied and found effective for the treatment of post-herpetic neuralgia and diabetic neuropathy.^1^ Carbamazepine has antineuralgic and muscle-relaxant properties and may depress synaptic transmission by limiting sodium-ion influx.^2^ Its exact mechanism for pain relief is unknown. Neither agent has been shown to be efficacious for the treatment of chronic low back pain, and therefore cannot be recommended. Pregabalin works in a similar way to gabapentin. Evidence from studies shows that pregabalin, when added to other treatments for low back pain, may provide additional benefit.^2^ Other less studied antiepileptic drugs, including topiramate, lamotrigine, levetiracetam, phenytoin, sodium valproate, zonisamide, and tiagabine are used as second line agents for chronic pain. Systematic reviews from 2007 and 2014 did not find evidence to support the use of lamotrigine and levetiracetam respectively.^1^ A complete blood count and baseline liver function test should be obtained prior to starting patients on older anticonvulsants such as phenytoin, carbamazepine, and valproic acid. They should be followed for the first three weeks then periodically. Blood levels do not correlate with efficacy so doses are titrated based on response.

Systemic Steroids

Systemic steroids cannot be recommended as a treatment for low back pain, as there is no evidence that demonstrates a benefit from their use.^2^

Cannabis and Cannabinoids

Systematic reviews and meta-analyses of trials including multiple patient populations and formulations of cannabis and cannabinoids have found some evidence of efficacy for chronic pain.^1^ A 2017 meta-analysis of 27 RCTs and 3 observational cohort studies of plant-based use (smoked, ingested, or nabiximol spray, but not the synthetic drugs dronabinol and nabilone) for treatment of chronic pain concluded that there was limited low-strength evidence that cannabis might alleviate neuropathic pain in some patients, but insufficient evidence for other types of chronic pain.^1^

Topical Therapies

Small clinical trials exist involving the use of a lidocaine 5% patch for the treatment of low back pain. The largest trial (6-week, open-label, nonrandomized) with 77 patients documented that 58% of patients were satisfied or very satisfied with the lidocaine patch for treatment of low back pain.^2^ Patients with low back pain of varying severity wore between 1 and 4 patches daily. Assessments completed at 2 and 6 weeks documented improvement in pain intensity and pain interference with quality of life, with minimal to moderate side effects, mainly dizziness and rash. At present, with only minimal published literature available regarding the use of lidocaine 5% patches for the treatment of low back pain, this therapeutic option should be reserved for use when other options have failed to help the patient achieve optimal pain relief. These patches would not be an option in patients with hepatic disease, as these patients are at a higher risk of lidocaine toxicity due to their inability to metabolize the drug normally. Further research using the lidocaine 5% patch is warranted.

Capsaicin, a derivative of chili peppers is believed to work by depleting substance P. It is available as a cream (0.025% or 0.075%). A systematic review found it to have moderate to poor efficacy for treating both neuropathic and nociceptive pain.^1^ It may be helpful as an adjunct or in patients who have failed other treatment.^1^ A meta-analysis of four RCTs with 1,272 patients concluded that capsaicin 8% patches applied for 30 to 90 minutes provided greater pain relief than low-concentration topical capsaicin after 12 weeks (number needed to treat [NNT] = 7; 95% confidence interval [CI], 5 to 15).5 However, the 8% patch is irritating and likely to cause pain when applied. A trained clinician should pretreat the application site with topical anesthetic before affixing the patch.^5^

Topical NSAIDs provide some improvement in acute musculoskeletal pain.^1^ Evidence is limited for its use in treating chronic low back pain and neuropathic pain. A systematic review found topical diclofenac 1.5% solution to be effective in the treatment of knee pain due to osteoarthritis.^1^

Transcutaneous electrical stimulation (TENS) involves the application of electrical currents from a small battery-operated device to the skin via surface electrodes. The majority of TENS devices offer variable frequency, pulse duration, intensity and type of output (burst or continuous). Combinations of different stimulation parameters are used to produce four main modes of TENS: conventional (high frequency, short pulse, low intensity); acupuncture-like (low frequency, long pulse duration, high intensity); burst (high frequency trains of pulses delivered at a low frequency); and brief-intense (high frequency and long pulse duration pulses delivered at a high intensity).^1^ Conventional TENS produces paresthesia in the area under the electrodes whereas the production of muscle twitches is desirable with acupuncture like TENS. Research on TENS for pain relief has suffered from a lack of rigorous RCTs and systematic reviews have found variable and inconclusive results of efficacy in chronic pain management.^1^ Further evidence is required to determine efficacy, parameter specific effects and cost-effectiveness of TENS. Optimal stimulation parameters and treatment durations should be considered while interpreting the outcome of systematic reviews of TENS.

Sources Adapted From:

1. Rosenquist E. Overview of the Treatment of chronic non-cancer pain. In: Aronson M, Crowley M, Ed. *Up to Date*, Waltham, Mass, 2018 www.uptodate.com/contents/ Overview of the Treatment of chronic non-cancer pain. Accessed September 21, 2019.

2. Miller LS. Low Back Pain: Pharmacologic Management. *Prim Care Clinic Office Practice*. 2012; (39): 499–510.

3. Williams C, Maher C, Latimer J. Efficacy of paracetamol for acute low-back pain: a double-blind, randomized controlled trial. *The Lancet*. 2014; (384): 1586-1596.

4. Dowell D, Haegerich T, Chou R. CDC Guideline for Prescribing Opioids for Chronic Pain. *MMWR.* 2016; (65): 1-50.

5. Saguil A, Kane S, Mercado M. Herpes Zoster and Postherpetic Neuralgia: Prevention and Management. *American Family Physician*. 2017; 96(10):656-663.

TEAM BASED LEARNING INSTRUCIONS

**Definition**: Team-Based Learning is an evidence based collaborative learning teaching strategy designed around units of instruction, known as “modules,” that are taught in a three-step cycle: preparation, in-class readiness assurance testing, and application-focused exercise.

**Preparation before class**

Students must complete preparatory materials before a class or the start of the module. The reading assignment has been emailed to you as an attachment at the beginning for the clerkship.

**In-class Readiness Assurance Testing**

Readiness Assurance Test (RAT):  Students complete an individual readiness assurance test (IRAT), consisting of 5 multiple choice questions.  After choosing your individual answers, you will, as individual teams come up with your group answer and use the provided scratch cards (IF-AT cards), hoping to find a star that indicates a correct answer.

Appeals: Teams have the opportunity to do a written appeal of a MCQ they felt was poorly written, the answer was mistakenly coded, or their answer choice is better.

Instructor Feedback:  The instructor may review material from the RAT that students still feel are problematic.

**In-class application focused exercise**

The remainder of the session is taken up with the Group Application Activity that helps you learn how to apply and extend the knowledge that you have pre-learned and been tested on. All teams are given 5 multiple choice questions to answer and must arrive at a consensus to choose a “best” solution out of options provided. Teams then display their answer choice simultaneously question by question with the provided answer signs, and this will facilitate a classroom discussion between teams to explore the topic and the possible answers to the problem.
