# Supplementary material for: Introduction to Pain Management for Third-Year Medical Students Team-Based Learning Module
Source: MedEdPORTAL. 2021 Feb 11;17:11095. doi: 10.15766/mep_2374-8265.11095 (PMC7880255; doi:10.15766/mep_2374-8265.11095)
Supplement: Supplementary file 1 — Pain Management TBL Advance Preparation Resources.docxPain Management TBL iRAT.docxPain Management TBL gRAT Group Answer Form.docxPain Management TBL gRAT Answer Key.docxPain Management TBL Team Application.docxPain Management TBL Team Application Answer Cards.docxPain Management TBL Team Application Answer Key.docxPain Management TBL Appeals Form.docx [file mep_2374-8265.11095-s001.zip › B. Pain Management TBL iRAT.docx]

ATTENTION, STUDENTS: If you are accessing this material BEFORE it is used in your course, please do NOT read this document prior to the class session. An answer key is included in this module, which is designed to lead you through a learning experience that reinforces your knowledge of the content. Early review or dissemination of this material to others will diminish the learning opportunity and be considered academic misconduct.

TEAM BASED LEARNING PAIN MANAGEMENT MODULE

iRAT (individual readiness assessment test)

Name ______________________________

Date ______________________________

**1.** Which one of the following antiepileptic drugs does not have any evidence to support its use in treating chronic pain?

a. gabapentin

b. carbamazepine

c. pregabalin

d. levetiracetam

**2.** Which one of the following antiepileptics used in treating chronic pain does not require obtaining a baseline CBC/LFT before initiating?

a**.** phenytoin

b. valproic acid

c. carbamazepine

d. gabapentin

**3.** Which of the following antidepressants is least effective for treating chronic pain?

a**.** amitriptyline

b. duloxetine

c. fluoxetine

d. nortriptyline

**4.** Which of the following TCAs has the greatest anticholinergic effects.

a. amitriptyline

b. nortriptyline

c. doxepin

d. desipramine

**5.** You are treating a patient for spasticity from cerebral palsy. Which of the following is the most appropriate agent?

a. cyclobenzaprine

b. baclofen

c. methocarbamol

d. carisoprodol
