# Supplementary material for: Introduction to Pain Management for Third-Year Medical Students Team-Based Learning Module
Source: MedEdPORTAL. 2021 Feb 11;17:11095. doi: 10.15766/mep_2374-8265.11095 (PMC7880255; doi:10.15766/mep_2374-8265.11095)
Supplement: Supplementary file 1 — Pain Management TBL Advance Preparation Resources.docxPain Management TBL iRAT.docxPain Management TBL gRAT Group Answer Form.docxPain Management TBL gRAT Answer Key.docxPain Management TBL Team Application.docxPain Management TBL Team Application Answer Cards.docxPain Management TBL Team Application Answer Key.docxPain Management TBL Appeals Form.docx [file mep_2374-8265.11095-s001.zip › D. Pain Management TBL gRAT Answer Key.docx]

ATTENTION, STUDENTS: If you are accessing this material BEFORE it is used in your course, please do NOT read this document prior to the class session. An answer key is included in this module, which is designed to lead you through a learning experience that reinforces your knowledge of the content. Early review or dissemination of this material to others will diminish the learning opportunity and be considered academic misconduct.

TEAM BASED LEARNING PAIN MANAGEMENT MODULE

gRAT (group readiness assessment test)

Date ______________________________

**1.** Which one of the following antiepileptic drugs does not have any evidence to support its use in treating chronic pain?

a. gabapentin

b. levetiracetam

c. pregabalin

d. carbamazepine

Correct answer B:

Gabapentin and carbamazepine, both anticonvulsants, have been used to treat chronic low back pain and have been proved to be efficacious for the treatment of sciatica.4 Gabapentin has a high affinity for voltage-gated calcium channels, which may modulate the release of excitatory neurotransmitters that affect nociception.2 Gabapentin has primarily been studied and found effective for the treatment of post-herpetic neuralgia and diabetic neuropathy.1 Carbamazepine has antineuralgic and muscle-relaxant properties and may depress synaptic transmission by limiting sodium-ion influx.2 Its exact mechanism for pain relief is unknown. Neither agent has been shown to be efficacious for the treatment of chronic low back pain, and therefore cannot be recommended. Pregabalin works in a similar way to gabapentin. Evidence from studies shows that pregabalin, when added to other treatments for low back pain, may provide additional benefit.2 Other less studied antiepileptic drugs, including topiramate, lamotrigine, levetiracetam, phenytoin, sodium valproate, zonisamide, and tiagabine are used as second line agents for chronic pain. Systematic reviews from 2007 and 2014 did not find evidence to support the use of lamotrigine and levetiracetam respectively.1 A complete blood count and baseline liver function test should be obtained prior to starting patients on older anticonvulsants such as phenytoin, carbamazepine, and valproic acid. They should be followed for the first three weeks then periodically. Blood levels do not correlate with efficacy so doses are titrated based on response.1

**2.** Which one of the following antiepileptics used in treating chronic pain does not require obtaining a baseline CBC/LFT before initiating?

a**.** carbamazepine

b. valproic acid

c. gabapentin

d. phenytoin

Correct answer C:

A complete blood count and baseline liver function test should be obtained prior to starting patients on older anticonvulsants such as phenytoin, carbamazepine, and valproic acid. They should be followed for the first three weeks then periodically. Blood levels do not correlate with efficacy so doses are titrated based on response.

**3.** Which of the following antidepressants is least effective for treating chronic pain?

a**.** amitriptyline

b. duloxetine

c. fluoxetine

d. nortriptyline

Correct answer C:

Tricyclic antidepressants and serotonin norepinephrine reuptake inhibitors possess analgesic qualities while the evidence for the effectiveness of selective serotonin reuptake inhibitors is weaker.1 The mechanism of action is unclear since TCAs with the greatest effect on serotonin are the most effective while fluoxetine, a potent serotonin reuptake inhibitor has little to no effect on reducing pain.

**4.** Which of the following TCAs has the greatest anticholinergic effects.

a. amitriptyline

b. nortriptyline

c. doxepin

d. desipramine

Correct answer A:

While effective at treating pain, TCAs have various side effects depending on the agent. Side effects include anticholinergic, antihistaminergic (doxepin has the most antihistaminergic effects) and alpha-1 adrenergic receptor blockade, and cardiac effects (increasing intraventricular conduction, prolonged QT interval, and prolonged AV nodal conduction). Amitriptyline has the most potent anticholinergic effects and despiramine has the least and is thus the least sedating. Anticholinergic effects include dry mouth, orthostatic hypotension, constipation, and urinary retention.

**5.** You are treating a patient for spasticity from cerebral palsy. Which of the following is the most appropriate agent?

a. baclofen

b. methocarbamol

c. cyclobenzaprine

d. carisoprodol

Correct answer A:

Two categories of muscle relaxants exist. Antispastic agents (baclofen, tizanidine, dantrolene, and diazepam) carry an indication for spasticity related to injury to the central nervous system (ie, multiple sclerosis) and are not recommended for treatment of low back pain.2 The other category, antispasmodic agents (cyclobenzaprine, methocarbamol, carisoprodol, metaxalone) can be added to treatment plans for low back pain if patients do not respond adequately to first-line analgesics.
