# Supplementary material for: Introduction to Pain Management for Third-Year Medical Students Team-Based Learning Module
Source: MedEdPORTAL. 2021 Feb 11;17:11095. doi: 10.15766/mep_2374-8265.11095 (PMC7880255; doi:10.15766/mep_2374-8265.11095)
Supplement: Supplementary file 1 — Pain Management TBL Advance Preparation Resources.docxPain Management TBL iRAT.docxPain Management TBL gRAT Group Answer Form.docxPain Management TBL gRAT Answer Key.docxPain Management TBL Team Application.docxPain Management TBL Team Application Answer Cards.docxPain Management TBL Team Application Answer Key.docxPain Management TBL Appeals Form.docx [file mep_2374-8265.11095-s001.zip › E. Pain Management TBL Team Application.docx]

ATTENTION, STUDENTS: If you are accessing this material BEFORE it is used in your course, please do NOT read this document prior to the class session. An answer key is included in this module, which is designed to lead you through a learning experience that reinforces your knowledge of the content. Early review or dissemination of this material to others will diminish the learning opportunity and be considered academic misconduct.

TEAM BASED LEARNING PAIN MANAGEMENT MODULE

Team Application (tAPP)

***1.*** A 50 year old man with a history of hypertension, obesity, and chronic hepatitis C presents with a 3 day history of non-radiating low back pain after lifting boxes. The pain is stable and has been alternating local heating and ice with minor relief. Which of the following is the best next step?

a. acetaminophen 1000mg q 6

b. acetaminophen 1000mg q 6 prn

c. acetaminophen 500mg q 6

d. acetaminophen 500mg q 4

**2.** A 60 year old man with a history of osteoarthritis, BPH, and CAD presents with recurrent R knee pain. He has been using ice and elevating 3 times and day and taking maximum dose acetaminophen without benefit for a week. Which is the next best addition?

a. ibuprofen 800mg q 6

b. naproxen 250mg q 6

c. diclofenac 50mg q 8

d. nabumetone 1000mg BID

**3.** A 30 year old woman complains of neck pain after being rear ended 2 days ago. She is currently taking daily a low dose OCP, esomeprazole for gastritis, and amitriptyline for IBS. She had been applying IcyHot (topical menthol and camphor) 3 times a day, wearing a soft collar neck brace and taking acetaminophen 1000mg q 6 all of which help briefly. Which medication should be added next?

a. celecoxib 100mg BID

b. piroxicam 10mg BID

c. ketorolac 10mg q 6

d. meloxicam 7.5 mg BID

**4.**  A 42 year old man with spinal stenosis is currently taking morphine 30mg extended release BID with hydrocodone 7.5mg/acetaminophen 325 mg BID PRN pain. He cannot tolerate the itching from the morphine and you notice his liver function is worsening and would like to discontinue his acetaminophen as well as switch him to a different long acting agent. Using the conversion chart below which of the following is the most appropriate regimen?

| **Equianalgesic Doses of Opioid Analgesics** |  |  |
| --- | --- | --- |
| **Oral/Rectal Dose (mg)** | **Analgesic** | **Parenteral Dose (mg)** |
| 100 | Codeine | 60 |
| - | Fentanyl | 0.1 |
| 15 | Hydrocodone | - |
| 4 | Hydromorphone | 1.5 |
| 2 | Levorphanol | 1 |
| 150 | Meperidine | 50 |
| 15 | Morphine | 5 |
| 10 | Oxycodone | - |

a. oxycodone 10mg extended release BID/oxycodone 7.5 mg BID PRN

b. oxycodone 30mg extended release BID/oxycodone 5 mg BID PRN

c. oxycodone 20mg extended release BID/oxycodone 7.5 mg BID PRN

d. oxycodone 20mg extended release BID/oxycodone 5mg BID PRN

**5.** A 55 year old woman complains of left shoulder pain for 2 weeks after falling. She had rotator cuff surgery on the same shoulder 3 years ago. You order an ultrasound which shows soft tissue swelling without a tear. She has been alternating acetaminophen 1000mg and ibuprofen 800mg every 3-4 hours without benefit. She has a history of uterine fibroids, depression, and GERD. She takes fluoxetine 60 mg, trazodone 150mg, and famotidine 40mg a day. When switching to a different pain management regimen which of the following medications should be avoided?

a. tramadol 50mg 1-2 tablets q 6

b. oxycodone 5mg/acetaminophen 325mg 1-2 tablets q 6

c. lidocaine 4% solution apply to affected area TID

d. hydrocodone 5mg/acetaminophen 300mg 1-2 tablets q 6
