# Supplementary material for: Introduction to Pain Management for Third-Year Medical Students Team-Based Learning Module
Source: MedEdPORTAL. 2021 Feb 11;17:11095. doi: 10.15766/mep_2374-8265.11095 (PMC7880255; doi:10.15766/mep_2374-8265.11095)
Supplement: Supplementary file 1 — Pain Management TBL Advance Preparation Resources.docxPain Management TBL iRAT.docxPain Management TBL gRAT Group Answer Form.docxPain Management TBL gRAT Answer Key.docxPain Management TBL Team Application.docxPain Management TBL Team Application Answer Cards.docxPain Management TBL Team Application Answer Key.docxPain Management TBL Appeals Form.docx [file mep_2374-8265.11095-s001.zip › G. Pain Management TBL Team Application Answer Key.docx]

ATTENTION, STUDENTS: If you are accessing this material BEFORE it is used in your course, please do NOT read this document prior to the class session. An answer key is included in this module, which is designed to lead you through a learning experience that reinforces your knowledge of the content. Early review or dissemination of this material to others will diminish the learning opportunity and be considered academic misconduct.

TEAM BASED LEARNING PAIN MANAGEMENT MODULE

Team Application (tAPP)

***1.*** A 50 year old man with a history of hypertension, obesity, and chronic hepatitis C presents with a 3 day history of non-radiating low back pain after lifting boxes. The pain is stable and has been alternating local heating and ice with minor relief. Which of the following is the best next step?

a. acetaminophen 1000mg q 6

b. acetaminophen 1000mg q 6 prn

c. acetaminophen 500mg q 6

d. acetaminophen 500mg q 4

Correct answer C:

A typical dosing regimen for acetaminophen would be 1000 mg orally every 6 hours, with a maximum daily dose of 4000 mg. Maximum doses should be used for the shortest time possible to minimize occurrence of adverse events. The adverse effect of greatest concern with acetaminophen is liver toxicity. Data from surveillance programs show that in an 8-year period (1990–1998) there were around 56,000 visits to emergency rooms, 26,000 hospitalizations, and 458 deaths due to acetaminophen overdoses. Consumption of alcohol while using acetaminophen containing products increases the risk of liver toxicity. Patients who consume alcohol or have concurrent liver toxicity need to limit their daily acetaminophen intake to 2000 mg. Most students choose C recognizing the need for a dose reduction given the patients history of chronic hepatitis C. The other choices add up potentially to greater than 2000mg a day. The most common incorrect answer given is A with students citing 4000mg as the highest daily dose and thus maximizing pain control but without accounting for the patient’s liver disease. Students are given the choice of as needed dosing illustrating the option of total daily dose reduction.

**2.** A 60 year old man with a history of osteoarthritis, BPH, and CAD presents with recurrent R knee pain. He has been using ice and elevating 3 times and day and taking maximum dose acetaminophen without benefit for a week. Which is the next best addition?

a. ibuprofen 800mg q 6

b. naproxen 250mg q 6

c. diclofenac 50mg q 8

d. nabumetone 1000mg BID

Correct answer B:

Concern exists for the use of NSAIDs and subsequent cardiovascular toxicity, mainly myocardial infarction. According to the literature, the risk appears to be increased over that of nonusers. In general, for patients at increased risk of cardiovascular toxicities, ibuprofen, diclofenac, and meloxicam should be avoided. Naproxen is the drug of choice, as it appears that it does not increase the risk of cardiovascular events. Students most commonly recognize the need to avoid NSAIDs other than naproxen in patients with cardiovascular disease and choose B. The most common incorrect answer is A given that ibuprofen is the NSAID they are most familiar with but do not take into account the patient’s history of CAD.

**3.** A 30 year old woman complains of neck pain after being rear ended 2 days ago. She is currently taking daily a low dose OCP, esomeprazole for gastritis, and amitriptyline for IBS. She had been applying IcyHot (topical menthol and camphor) 3 times a day, wearing a soft collar neck brace and taking acetaminophen 1000mg q6 all of which help briefly. Which medication should be added next?

a. celecoxib 100mg BID

b. piroxicam 10mg BID

c. ketorolac 10mg q 6

d. meloxicam 7.5 mg BID

Correct answer A:

Gastrointestinal toxicity, also resulting from prostaglandin inhibition, occurs at a rate of 1% to 2% of all NSAID use. Cyclooxygenase-2 inhibitors may have fewer gastrointestinal toxicities in comparison with traditional NSAIDs. Students typically choose A noting celecoxib being the least GI toxic given its different mechanism of action than the rest of the choices. When another answer is chosen it is typically B or C citing their maximum daily dosage listed as being more effective for pain control than answer D, meloxicam, which is listed at half the maximum daily dose.

**4.**  A 42 year old man with spinal stenosis is currently taking morphine 30mg extended release BID with hydrocodone 7.5mg/acetaminophen 325 mg BID PRN pain. He cannot tolerate the itching from the morphine and you notice his liver function is worsening and would like to discontinue his acetaminophen as well as switch him to a different long acting agent. Which of the following is the most appropriate regimen?

a. oxycodone 10mg extended release BID/oxycodone 7.5 mg BID PRN

b. oxycodone 30mg extended release BID/oxycodone 5 mg BID PRN

c. oxycodone 20mg extended release BID/oxycodone 7.5 mg BID PRN

d. oxycodone 20mg extended release BID/oxycodone 5mg BID PRN

Correct answer D:

| **Equianalgesic Doses of Opioid Analgesics** |  |  |
| --- | --- | --- |
| **Oral/Rectal Dose (mg)** | **Analgesic** | **Parenteral Dose (mg)** |
| 100 | Codeine | 60 |
| - | Fentanyl | 0.1 |
| 15 | Hydrocodone | - |
| 4 | Hydromorphone | 1.5 |
| 2 | Levorphanol | 1 |
| 150 | Meperidine | 50 |
| 15 | Morphine | 5 |
| 10 | Oxycodone | - |

Students typically choose D which involves a 1 to 1.5 ratio of dosage adjustment. This question typically took the longest for groups to answer. Noting the difficulty, the conversion chart from the pre-reading materials was then included in the question stem resulting in groups then taking the same amount of time answering it as the others.

**5.** A 55 year old woman complains of left shoulder pain for 2 weeks after falling. She had rotator cuff surgery on the same shoulder 3 years ago. You order an ultrasound which shows soft tissue swelling without a tear. She has been alternating acetaminophen 1000mg and ibuprofen 800mg every 3-4 hours without benefit. She has a history of uterine fibroids, depression, and GERD. She takes fluoxetine 60 mg, trazodone 150mg, and famotidine 40mg a day. When switching to a new pain medication regimen which of the following drugs should be avoided?

a. tramadol 50mg 1-2 tablets q 6

b. oxycodone 5mg/acetaminophen 325mg 1-2 tablets q 6

c. lidocaine 4% solution apply to affected area TID

d. hydrocodone 5mg/acetaminophen 300mg 1-2 tablets q 6

Correct answer A:

Major side effects, occurring at a rate greater than 10%, associated with tramadol include dizziness, nausea, sedation, constipation, and headache. Tramadol use at or above recommended doses has also been associated with seizures. When combined with tramadol, patients receiving serotonin reuptake inhibitors (SSRIs), tricyclic antidepressants, monoamine oxidase inhibitors, neuroleptics, or other opioids have an even higher potential for occurrence of seizures. Tramadol has a large number of drug interactions, many involving the cytochrome P450 enzyme system (2B6, 2D6, and 3A4).

Students typically correctly choose A citing the increased potential for seizures when tramadol is combined with SSRIs. B and D are common incorrect answers. The reasoning being the same for both that is safer to use alternative pain medications before prescribing an opioid.
