# Supplementary material for: Introduction to Pain Management for Third-Year Medical Students Team-Based Learning Module
Source: MedEdPORTAL. 2021 Feb 11;17:11095. doi: 10.15766/mep_2374-8265.11095 (PMC7880255; doi:10.15766/mep_2374-8265.11095)
Supplement: Supplementary file 1 — Pain Management TBL Advance Preparation Resources.docxPain Management TBL iRAT.docxPain Management TBL gRAT Group Answer Form.docxPain Management TBL gRAT Answer Key.docxPain Management TBL Team Application.docxPain Management TBL Team Application Answer Cards.docxPain Management TBL Team Application Answer Key.docxPain Management TBL Appeals Form.docx [file mep_2374-8265.11095-s001.zip › H. Pain Management TBL Appeals Form.docx]

| **Team Number:** |
| --- |
| **Question Number being Appealed:** |
| **Argument and Evidence to Support Appeal:** |

TEAM BASED LEARNING

PAIN MANAGEMENT

If your team would like to make an appeal to one of the questions, provide evidence below citing the source of ambiguity in the question or pre-participation materials and suggest less ambiguous wording.
